# A Rare Case of *Pseudomonas aeruginosa*-Associated Diarrhea in a Cirrhotic Patient

**DOI:** 10.1155/2024/8238951

**Published:** 2024-08-16

**Authors:** Malcolm Chapman, Atul Lodh, Joven Tristeza, Alexander Yang

**Affiliations:** University of Alabama Birmingham Department of Internal Medicine, Birmingham, AL, USA

## Abstract

While rare, cases of community-acquired *Pseudomonas aeruginosa* (*PsA*) enterocolitis have been reported and are associated with recent antibiotic use and immunocompromised hosts. Here we present a 29-year-old immunocompetent female with newly diagnosed alcoholic cirrhosis that presents with bloody diarrhea and was found to have *PsA* in her stool culture. This case is unique as our patient does not have a history of recent antibiotic use or prior history of immunosuppression. However, immune dysfunction in cirrhosis results in defects in both innate and acquired immunity, thus predisposing our patient to *PsA*-associated diarrhea. Overall, this case showcases that, while considered immunocompetent, cirrhotic patients are predisposed to rare infections such as *PsA* in the community due to defects in their immune system.

## 1. Introduction

*Pseudomonas aeruginosa* (*PsA*) is an opportunistic Gram-negative pathogen commonly associated with enterocolitis in children and nosocomial respiratory, genitourinary, and bloodstream infections [1]. While rare, cases of community-acquired *PsA* enterocolitis have been reported and are associated with recent antibiotic use and immunocompromised hosts. In the absence of the above risk factors *PsA* enterocolitis is extremely rare similar to fungal infections as immunocompetent hosts are able to prevent overt infection of the colonized bacteria [2, 3]. Here, we present a 29-year-old female with decompensated alcohol-associated cirrhosis complicated by alcoholic hepatitis who experienced acute bloody diarrhea secondary to community-acquired *PsA* colitis.

## 2. Case Presentation

A 29-year-old female presented to the emergency department with progressively worsening abdominal swelling over 4 to 6 months and recent bloody diarrhea over the last week. Her diarrhea was described to be large voluminous watery diarrhea that was soaked in blood. She had no prior past medical history and took no medications. Of note, she also was not recently on any antibiotics or steroids. Her workup on admission included a CMP that showed elevated transaminases AST 237, ALT 40, total bilirubin 19.3, direct bilirubin 14.2, indirect bilirubin 5.1, albumin 2.9 along with electrolyte abnormalities sodium of 128, potassium of 2.3, chloride 88. CBC was remarkable for leukocytosis WBC of 14,500 with 76% neutrophils, hemoglobin of 9 gm/dL, and platelets of 170. Right upper quadrant ultrasound imaging displayed hepatomegaly with cirrhotic morphology. Patient was started on diuretics for her abdominal swelling, with etiology of her cirrhosis suspected to be secondary to significant alcohol use. Initial infectious workup was only notable for positive rhinovirus/enterovirus. Stool culture was acquired during her hospitalization which came back positive for *Pseudomonas aeruginosa* and negative for C. difficile, *Salmonella*, *Shigella*, *Vibrio*, *Yersinia*, and *E. coli* O157: H7. The antibiogram showed susceptibility to ciprofloxacin, cefepime, and piperacillin. She was started on ciprofloxacin 500 mg twice daily for a 10-day total course, with slow resolution of her diarrhea by the day of discharge. A repeat stool culture was not done as patient had resolution of her symptoms.

## 3. Discussion

*PsA* rarely causes diarrhea and can be isolated in about 4% of asymptomatic people [4]. *PsA*-associated diarrhea is more often seen in pediatric populations [2, 5, 6], adult patients with immunodeficiency [2], and adult patients with recent antibiotic use [2, 4, 7–9]. Recent estimates show that *PsA* accounts for about 1% and 5.6% of hospital-acquired diarrhea [9, 10].

Patients with cirrhosis are considered immunocompromised and have heightened vulnerability to such pathogens due to immune cell dysfunction and impaired gut barrier function, resulting in bacterial translocation that promotes microbial dysbiosis [11]. This immune dysfunction in cirrhotic patients is known as cirrhosis-associated immune dysfunction (CAID) and results in alteration of both innate and acquired immunity [12]. Specifically, patients with alcoholic-related cirrhosis have been shown to have broad defects in T cells and hyperactivity of B cells resulting in increased hepatic production of proinflammatory cytokines and chemokines, thus predisposing our patient for a rare community-acquired diarrhea secondary to *PsA* colitis [13].

Since the presentation of *PsA* infection as diarrhea is rare, there are currently no consensus guidelines on treatment. One study in neonates showed that treatment with antipseudomonal antibiotics did not influence rapidity of clearance in stool cultures or alleviation of symptoms [6]. In adults, one study only recommended anti-pseudomonal therapy if there were signs of a disseminated infection [7]. However, this recommendation was made as an expert opinion since the study did not compare outcomes when the patients were treated with antibiotics [8]. Future prospective randomized control studies of adults with *PsA*-associated diarrhea would help provide more evidence-based guidance on treatment options.

Besides our reported case, there are only two other case reports of *PsA*-associated diarrhea occurring in an immunocompetent patient from the United States: a long-term critically ill hospitalized patient treated with broad-spectrum antibiotics along with antifungals prior to having their course complicated by *PsA* diarrhea [14], and an ambulatory patient with history of alcohol abuse who recently finished a course of antibiotics [15]. Interestingly, our patient had no recent antibiotic exposure. Furthermore, the other reported patients were aged 81 and 53, respectively, whereas our patient was a much younger 29-year-old female. In both case reports, the patients were treated with ciprofloxacin with resolution of diarrhea similar to our case.

## 4. Conclusion

Here, we present a case of community-acquired *PsA*-associated diarrhea in a young female with decompensated alcohol-associated cirrhosis that otherwise did not display typical risk factors we associate with immunodeficiency. Given its rarity, our case contributes to the growing literature of *PsA*-associated diarrhea in otherwise seemingly immunocompetent patients. Cirrhosis has known pathophysiologic mechanisms that explain immune dysfunction. We use this case to highlight the importance of providers to recognize *PsA*-associated diarrhea in adults as a true, albeit uncommon, gastrointestinal infection and cirrhosis as a form of immunodeficiency that can predispose to develop this infectious process.

## Data Availability

The data used to support the findings of this study are included within the article.
